# Ligand-biased ensemble receptor docking (LigBEnD): a hybrid ligand/receptor structure-based approach

**DOI:** 10.1007/s10822-017-0058-x

**Published:** 2017-09-08

**Authors:** Polo C.-H. Lam, Ruben Abagyan, Maxim Totrov

**Affiliations:** 1 Molsoft L.L.C., 11199 Sorrento Valley Road, S209, San Diego, CA 92121 USA; 2 Skaggs School of Pharmacy and Pharmaceutical Sciences, University of California San Diego, La Jolla, CA 92093 USA

**Keywords:** Ligand docking, Receptor flexibility, Atomic property fields, ICM, D3R

## Abstract

Ligand docking to flexible protein molecules can be efficiently carried out through ensemble docking to multiple protein conformations, either from experimental X-ray structures or from in silico simulations. The success of ensemble docking often requires the careful selection of complementary protein conformations, through docking and scoring of known co-crystallized ligands. False positives, in which a ligand in a wrong pose achieves a better docking score than that of native pose, arise as additional protein conformations are added. In the current study, we developed a new ligand-biased ensemble receptor docking method and composite scoring function which combine the use of ligand-based atomic property field (APF) method with receptor structure-based docking. This method helps us to correctly dock 30 out of 36 ligands presented by the D3R docking challenge. For the six mis-docked ligands, the cognate receptor structures prove to be too different from the 40 available experimental Pocketome conformations used for docking and could be identified only by receptor sampling beyond experimentally explored conformational subspace.

**Electronic supplementary material:**

The online version of this article (doi:10.1007/s10822-017-0058-x) contains supplementary material, which is available to authorized users.

## Introduction

Consideration of protein flexibility is important in accurate ligand docking and effective virtual ligand screening (VLS) [1]. Numerous extensive benchmarking tests of various docking methods [2–11] in re-docking to cognate receptors have been reported [12–15] but the success in such benchmarks may not be representative of real-life performance in docking of novel ligands [16].

It has been shown that, for example, docking a co-crystallized ligand back to its cognate protein structure (self-docking) can be achieved with up to 90% success rate [17]; but when only a single protein structure is available for docking of different ligands, the success rate can drop to less than 50%, indicating that subtle side-chain or backbone rearrangements may prevent a ligand from docking into its native pose [18] This is especially problematic for protein with high backbone flexibility, and even a small backbone movement can affect multiple side-chain’s conformations.

Various efforts have been made in the past to solve this flexible protein–ligand docking problem. On one end of the spectrum, fully flexible protein–ligand docking simulation using molecular dynamics methods have been proposed [19, 20]. While this method appears to imitate the dynamics of protein–ligand interaction in reality, the high computational time requirements have limited its scope in VLS, when millions of compounds need to be evaluated, each requiring separate simulations for individual poses. Moreover, in the context of ligand docking when the native pose is unknown, the selection of correct pose from multiple plausible protein–ligand complexes requires a full energy function that takes into account the protein folding energy, ligand strain, protein–ligand interaction, desolvation penalty, etc. In this case, the simple docking problem is essentially transformed into a full-blown protein folding energy calculation, with the complication of a bound ligand.

On the other end of the spectrum, rigid receptor–flexible ligand docking can generally be completed in a matter of seconds or minutes. Its drawback, the lack of protein flexibility, has been addressed through ensemble docking–docking to multiple different protein conformations [21]. Ensemble docking treats the flexible protein as multiple discreet states instead of the continuously varying states in a fully flexible protein–ligand simulation, thus simplifying the full conformational search and energy evaluation problem. The success of ensemble docking hinges on the availability and selection of multiple complementary protein conformations [22]; this again can be broken down into two different challanges:

1. When a flexible protein’s known conformations are inadequate for correctly docking all known ligands, additional plausible protein conformations need to be generated. We have proposed in the past methods such as Dual Alanine Scanning and Refinement (SCARE) [23] and Ligand-guided Backbone Ensemble Receptor Optimization (ALiBERO) protocols [24]. The SCARE method is useful when side chain rearrangement followed by small backbone minimization is sufficient in generating alternative conformations. The ALiBERO method, on the other hand, can be used when significant backbone movement is needed [25]. The computational time for subsequent docking into multiple conformations can be further reduced through the use of 4D-grid docking method [26].
2. However, when a flexible protein has many known conformations in the Protein Data Bank (PDB) [27], or if conformation generation methods such as the SCARE and ALiBERO generate too many possible conformations, we are presented with a different problem not unlike the one faced by molecular dynamics method, namely, which one of the multiple ligand docking poses and receptor conformations is correct? Previous experience in ensemble docking shows that the initial improvement in docking pose accuracy going from single conformation to a handful of conformations is quickly offset by the introduction of false positive poses as more protein conformations are added [17]. Using all available conformations often lead to poor results as a ligand docked in a wrong pose/receptor conformation can incidentally give a better docking score than the correct/native pose as the number of conformations increases.

Practically, ensemble docking requires a compromise between two ‘pitfalls’ where either (I) no near-native ligand poses can be found because all receptor conformations in the ensemble fit the native pose too poorly; or (II) too many alternative conformations are generated, crowding out the near native pose with false positives of the scoring function. A useful strategy to address both pitfalls is to incorporate ligand structural data (when available) into simulations: on the one hand, it can be used to direct docking towards poses that resemble ligands in the available complex crystal structures, so that imperfections of the pocket fit can be overcome; on the other hand, scoring function for final pose ranking can be also biased towards poses resembling experimentally determined structures.

In the current study, we were presented with a challenge: docking of farnesoid X receptor (NR1H4) ligands not previously co-crystallized in the PDBs. We developed a new hybrid ligand/receptor structure-based docking and pose selection method Ligand-Biased Ensemble Docking (LigBEnD), by incorporating the atomic property field (APF) method [28] into structure-based ensemble docking. The ligand-based APF method has previously been shown to be a complementary alternative to docking, especially in the case when protein flexibility is not fully accounted for by the available protein conformations [29] For one family of the NR1H4 compounds, the use of Molsoft ICM docking score alone was adequate in predicting the correct poses. For other families of compounds in which the docking score does not unambiguously identify the correct poses, a composite score that combines the ICM docking score with APF similarity score proves to be helpful.

This new hybrid method assumes the following: (1) Compounds that are similar to co-crystallized ligands are likely to bind in a similar pose. (2) Compounds that are chemically dissimilar to co-crystallized ligands might share similarity in the properties of atoms occupying the same 3D space. (3) Compounds belonging to the same chemical class should have consistent, similar poses. The comparisons of poses between docked ligands and co-crystallized ligands, as well as among the docked ligands, are achieved through the use of ICM’s APF distance calculation [30].

## Methods

### Receptor grid potential maps preparation

All protein structures used came from the Pocketome entry for farnesoid X receptor (NR1H4_HUMAN_257_485) [31]. Pocketome is a large pocket-centric collection of protein–ligand complexes originated from the PDB, each Pocketome entry is organized around a particular ligand pocket (i.e. PDB structures of the same protein may be present in different Pocketome entries if, for instance, ortho- and allo-steric pockets exist) from PDB entries of a single Uniprot entity. Pockets are optimally pre-aligned/superimposed, making Pocketome entries convenient starting point for ensemble docking. Pocketome entries also include different biologically equivalent chains in the crystal structure so that conformational variations observed within single crystal form are incorporated in the resulting ensembles. The NR1H4_HUMAN_257_485 entry consists of 40 different protein chains/conformations originated from 28 PDB entries of NR1H4_HUMAN. Each of the 40 protein conformations were converted into an ICM object using the standard ICM procedure: [32, 33].

The protein atoms were assigned to the correct atom types and charge based on a modified ECEPP force field [34], the ligand atoms were assigned based on the modified Merck force field (MMFF94) [35]. Missing hydrogen atoms and zero-occupancy heavy atoms were added. Side chains with added atoms and polar hydrogen atoms, or side chains with multiple tautomeric or rotational conformations such as glutamine, asparagine, histidine, were sampled and optimized in the presence of the co-crystallized ligands. The co-crystallized ligand in the PDB entry was then removed and processed separately (*vide infra*) as an APF ligand template.

The ligand-binding pocket was defined by protein residues within 5 Å of the co-crystallized ligand. Five grid potential maps for a 3D-box that encapsulate the ligand-binding pocket residues were calculated with a 0.5 Å grid spacing. These maps represent electrostatics, hydrophobicity, hydrogen bonding, and the soft van der Waals potentials for hydrogens and for heavy atoms.

### Co-crystallized ligand atomic property field (APF) grid maps preparation

The co-crystallized ligand separated during the receptor grid map preparation was converted to APF grid maps to guide and accelerate the docking process: [28] each atom of the co-crystallized ligand is represented by a vector of seven components, corresponding to seven physiochemical properties: hydrogen bond donor, hydrogen bond acceptor, sp^2^ hybridized, lipophilic, size, charged, and electronegative/electropositive. Seven grid maps were then calculated to represent the property fields of the co-crystallized ligand in 3D space as a total of Gaussian property fields from each ligand atom. For any ligand atom, its APF score or pseudo energy is the dot product of its property vector and the APF potential at that space. Thus the APF method allows one to: (1) optimally (in the sense of matching physicho–chemical atomic properties) fit any ligand to the grid representation of co-crystallized ligands through Monte Carlo sampling of internal variables followed by energy minimization. (2) Calculate the APF ‘interaction’ energy of any two ligand poses (for the same or different ligands), giving a measure of chemical 3D similarity. This similarity measure is topology-independent, i.e. doesn’t require or imply any specific atom-to-atom or bond-to-bond correspondence.

### Ligand preparation

The structures of the compounds were obtained from the assessment organizer (D3R) and converted into 2D drawings and processed in ICM: The formal charge of each atom was set using ICM’s pKa prediction model at pH 7. Stereochemistry and hydrogen atoms were assigned accordingly. Each atom was assigned MMFF94 force field atom type and partial charge. The 2D ligand was then converted to 3D, its rotational bonds sampled and all atoms minimized in the Cartesian coordinates in the absence of the receptor maps as the starting ligand conformation for docking.

### Docking ligand to the receptor grid maps and co-crystallized ligand template APF maps

Each ligand was docked to each of the protein conformations, represented by its receptor grid maps and co-crystallized ligand template APF grid maps, using the standard ICM protein–ligand docking procedure [36]. ICM ligand docking uses a biased probability Monte Carlo (BPMC) with local gradient minimization to optimize the docked ligand’s internal variables, including 6 positional variables and all freely rotatable bonds. Random moves were made to these variables, followed by energy minimization in the grid map representation of the receptor and the APF grid map of co-crystallized ligand. Multiple conformations of the ligand were stored and clustered by atomic RMSD (<2 Å) during simulation to ensure diversity of ligand poses. A docking ‘effort’ setting of 10 was used, which dictates the length of simulation and total number of energy minimization steps. At the end of each docking simulation, the 10 best conformations for each ligand were stored according to the combined receptor grid and co-crystallized ligand APF grid energies. They were re-evaluated using ICM’s standard VLS docking score *S*_*Dock*_ which is a GBSA/MM-type scoring function augmented with a directional hydrogen bonding term [17].

For the initial docking of the 36 compounds, each compound was docked to each of the 40 available PDBs from the Pocketome entry NR1H4_HUMAN_257_485, in 2 independent runs, both of which employed the ligand APF bias. The single best solution of each independent run was used for further processing and final pose selection. Each compound produced 40 × 2 = 80 poses.

### Post-docking processing and pose selection

In addition to the standard ICM VLS docking score, each ligand pose’s APF similarity to the co-crystallized ligand in the corresponding protein conformation was also calculated. The APF similarity score *S*_*APF*_ *(m,n)* between any two ligand poses m and n can be defined by: [30]

$${S_{APF}}(m,n)=\frac{{2{E_{APF}}(m,n)}}{{{E_{APF}}(m,m)+{E_{APF}}(n,n)}}$$

where *E*_*APF*_ *(m,m)* and *E*_*APF*_ *(n,n)* are the APF self ‘energy’ of ligand pose m and ligand pose n; *E*_*APF*_ *(m,n)* is the cross APF ‘energy’ between ligand poses m and n. A composite score *S*_*Comp*_ combing the ICM docking score *S*_*Dock*_ and ligand APF similarity score *S*_*APF*_ for each docking pose was simply:

$${S_{Comp}}={S_{Dock}}~ \times \;{S_{APF}}$$

No further optimization of the weight of *S*_*APF*_ relative to *S*_*Dock*_ was attempted in the current study.

### Pose consistency analysis within compound families

To classify the compounds, we first calculated the 2D fingerprint Tanimoto distance for each pair of compounds, and clustered them at a distance cutoff of 0.42 into four major families of compounds and six singletons which have no obvious similar neighbour. Within each chemical family, 40 × 2 poses for each ligand were pooled together and clustered using pairwise APF distance *D*_*APF*_ *(m,n)* between any two poses, according to the formula:

$${D_{APF}}\left( {m,n} \right)=1 - {S_{APF}}(m,n)$$

where *S*_*APF*_ *(m,n)* is the APF similarity score between pose m and pose n, defined before. The pairwise distances between poses were used to cluster different poses at an APF distance cutoff of 0.4. Note that for each chemical family, there are multiple pose clusters; each pose cluster can contain multiple similar poses from the same compound or from different compounds.

For each compound, one pose was selected from each pose cluster based on the best composite score S_Comp_. The top 5 ranked poses were submitted to the D3R assesment organizer as our predicted poses.

### Post-challenge evaluation and additional simulations

Upon the release of the 36 X-ray structures, we evaluated the ligand RMSD by the following method: For each ligand pose, the C_α_ atoms of protein conformation used for docking within 7 Å of the docked ligand were superimposed with the corresponding atoms of the X-ray structure. All heavy atoms of the ligand were used to calculate the RMSD between the predicted pose and the pose of the co-crystallized ligand.

We carried out additional docking for six mis-docked compounds to each of the 36 X-ray structures, each compound in two independent runs, one with APF bias from the co-crystallized ligand, one without. For each ligand in each run, the top 10 poses were retained and evaluated by ICM docking score and APF similarity score calculations. Each ligand produced 720 poses. From the previous docking results (using the 40 Pocketome conformations), the top 10 docking poses for each of the two independent runs, both with ligand APF bias, were extracted and combined with the new docking poses to generate a total of 1520 poses for each compound.

We also carried out SCARE simulations for six mis-docked compounds, starting with each of the 40 Pocketome conformations, using a modified, 4D version of the published settings [23]. The original SCARE protocol systematically mutated pairs of ligand pocket residues into alanine, docked the ligand into each modified pocket version to obtain a docking pose, then place these poses into original explicit receptor and perform side chain refinement and energy minimization. In the 4D version, the alanine substituted ‘conformations’ were combined into a single set of ‘4D’ receptor grid maps and single ‘4D’ docking run was performed. ‘4D’ grids store potentials generated from multiple receptor states as different layers in the fourth grid dimension [26]. In ‘4D’ docking runs, in addition to regular MC steps that change ligand position or conformation, grid ‘4D layer’ switch steps would effectively change receptor configuration. This ‘4D’ approach allowed us to accelerate and simplify ligand docking to different truncated forms of the pocket. For each of the ligand–protein conformation SCARE run, the top 40 poses were retained and refined by fully flexible side chain sampling/minimization. Each compound produced 40 × 40 = 1600 poses. The ICM docking score, APF similarity to the co-crystallized ligand in the initial protein conformation, and the RMSD from the final X-ray structures were calculated.

### Software and hardware

All calculations, including receptor and ligand preparation, grid potential map calculations, docking simulations, ICM docking score, APF similarity score, ligand pose clustering, and RMSD calculations, were carried out using ICM 3.8–6 (Molsoft LLC, San Diego, CA). The docking simulations and ICM docking score calculations were performed on a Linux cluster of 20 8-core (2×Intel Xeon E5620) compute nodes.

## Results and discussion

In the current docking assessment, we were given the challenge of docking 102 farnesoid X receptor (NR1H4) ligands, for the first 36 of which a co-crystallized structure was released after the end of the challenge. Our analysis will focus on these 36 compounds: the best score to use in pose selection and the comparison of the predicted poses versus the correct poses in the released structures. We first analyzed the chemical diversity of the 36 ligands by calculating the 2D fingerprint Tanimoto distance for each pair of compounds, and clustered them at a distance cutoff of 0.42 into four major families of compounds and six outliers which have no obvious similar neighbor. Distance cutoff was chosen so that compounds with common substructure core were grouped into one cluster.

**Fig. 1:**
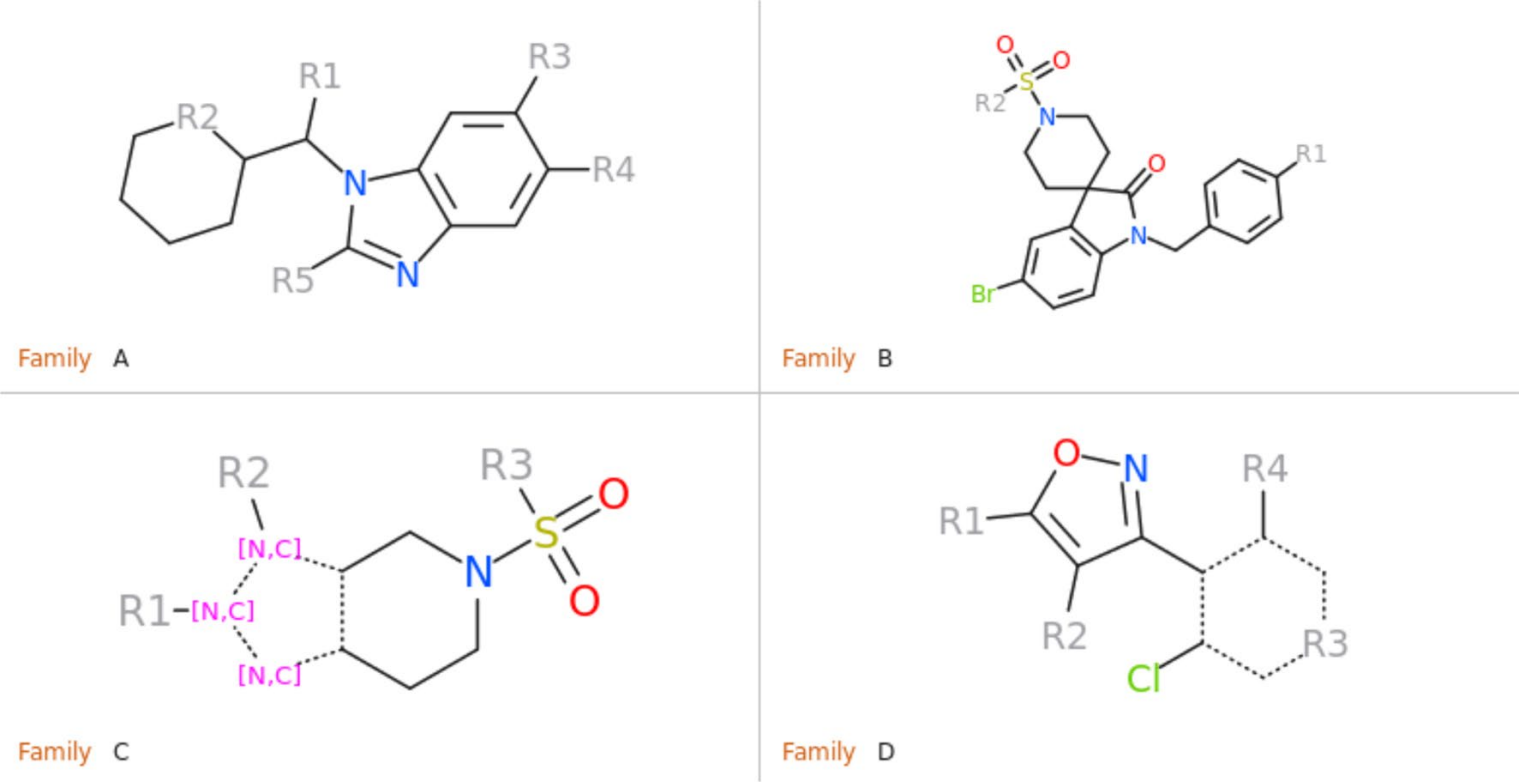
Common scaffolds for the four major families of compounds. Family C can be further divided into two smaller subgroups with either a 4,5,6,7-tetrahydro-1*H*-pyrrolo[2,3-c]pyridine or 4,5,6,7-tetrahydro-3*H*-pyrazolo[4,3-c]pyridine ring

**Fig. 2:**
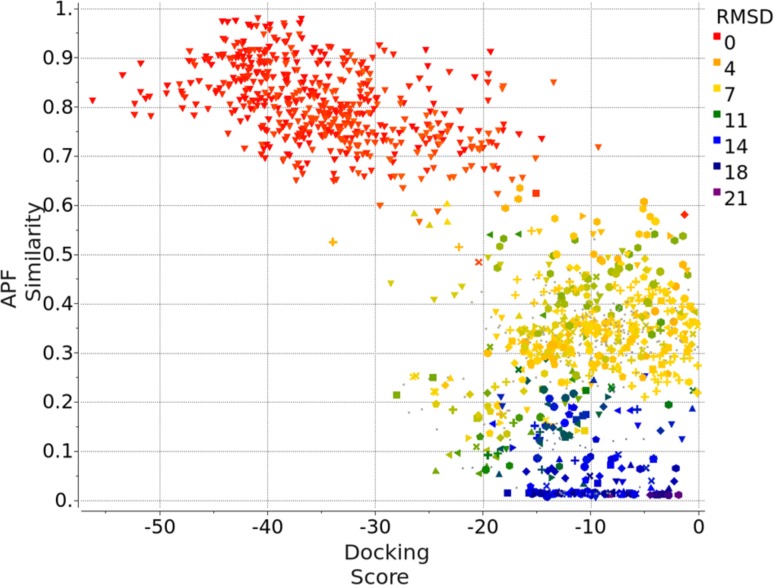
APF similarity score versus ICM Docking Score for 21 Family A compounds containing the benzimidazole core, docked to 40 protein conformations in Pocketome entry NR1H4_HUMAN_257_485, each in two independent runs: each compound generated 80 poses. Only the poses that have ICM docking score <0 are shown here. The pairwise APF distance between each pose were calculated, the poses were then clustered at an APF distance cutoff of 0.4. Poses belonging to the same cluster are represented by the same shape in the plot. After the release of the X-ray structure, each predicted pose’s RMSD to the correct pose was calculated and shown in *color gradient* according to the RMSD value

Compound Family A (see Fig. 1) is the largest family with a common benzimidazole ring at its core, containing 21 members out of the first 36 compounds, and 47 members out of the full set of 102 compounds. Each compound was docked to each of the 40 protein conformations in the Pocketome entry NR1H4_HUMAN_257_485 in two independent runs, both runs were carried out in the presence of that protein conformation’s co-crystallized ligand, in the form of APF ligand template grid map. The best docking pose for each of the independent run was re-evaluated by ICM’s standard docking score. In addition, we calculated the APF similarity between the docked ligand’s pose and the co-crystallized ligand’s pose. Figure 2 is the plot of APF similarity to co-crystallized template ligand versus ICM docking score for all the poses with ICM docking score below zero. For each pair of predicted poses, we evaluated pose similarity using the APF distance and clustered all the poses at an APF distance cutoff of 0.4. We noticed that all the poses with the most negative ICM docking score and highest APF similarity score belong to a single cluster of poses. This was encouraging as we expected compounds belonging to the same chemical class should have a similar docking pose. We also noted that some compounds can produce alternative docking poses with ICM docking score as low as −34, comparable with some of the members in the “correct” pose cluster. This is in line with our previous observations that, as we increase the number of protein conformations used in ensemble docking, false positives introduced start to offset the benefit of increased conformational diversity—wrong ligand poses can incidentally produce a better docking score than correct ligand poses in a slightly incompatible protein conformation. However, these incorrect poses typically do not resemble cognate ligand poses and therefore have low S_APF_. Therefore, we decided to construct a new composite score S_Comp_, which is the simple product of ICM docking score S_Dock_ and APF similarity score to co-crystallized ligand S_APF_, the compounds with the best (most negative) S_Comp_ are located in the top left quadrant of the APF Similarity versus Docking Score plot. Therefore S_Comp_ reflects both the quality of ligand-receptor fit as well as similarity of the pose to the cognate ligand X-ray structure pose for a given receptor conformation. We selected the top pose for each ligand using S_Comp_. After the release of the X-ray structures at the end of the challenge, the RMSD between the prediction and the correct answer for each compound was calculated and represented in color gradient in Fig. 2. The poses with the most negative S_Comp_ did correspond to the lowest RMSD and most accurately docked compounds.

**Fig. 3:**
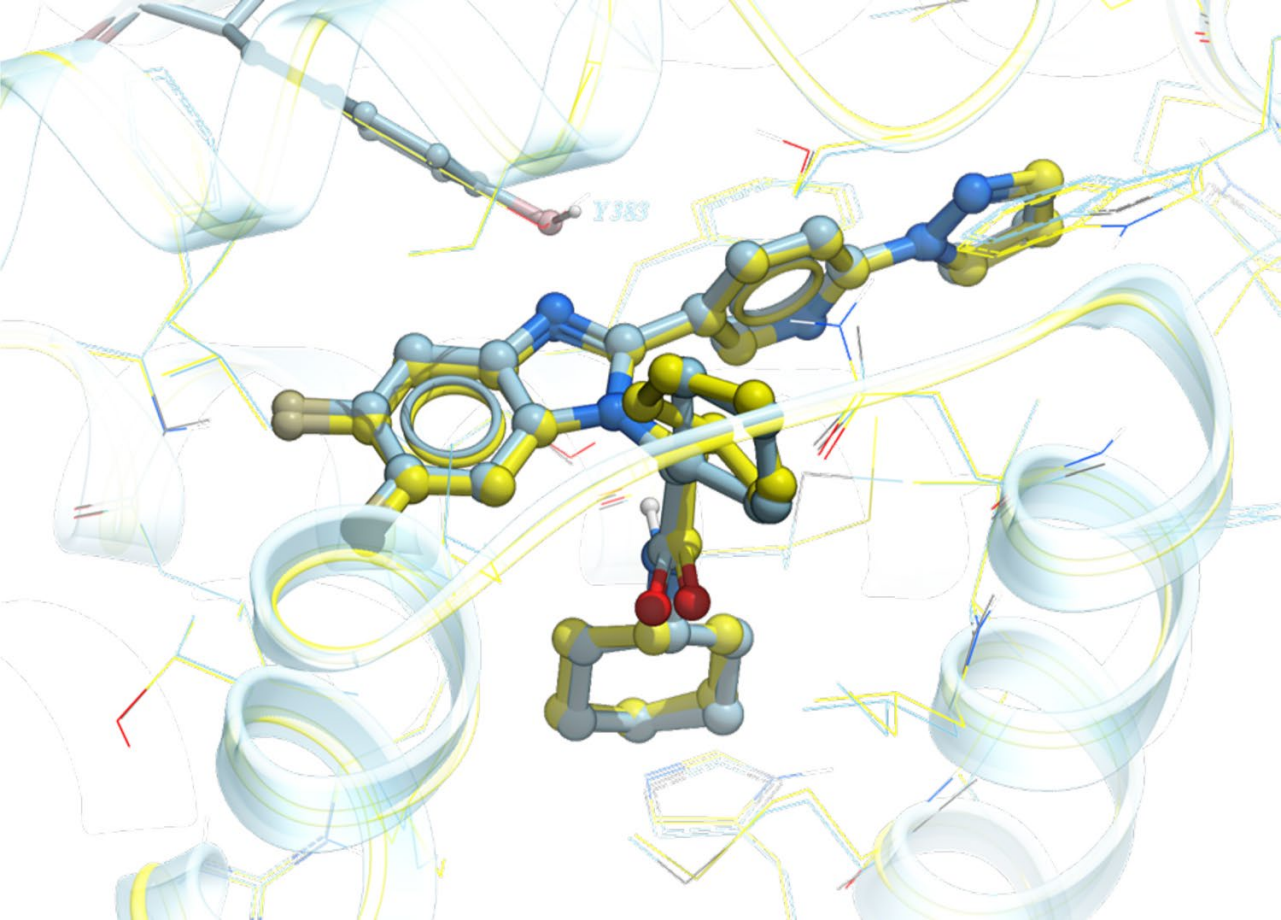
Predicted docking pose of FXR_26 (*blue*) from family A versus the co-crystallized structure released (*yellow*). All residues within 5 Å of the ligand are displayed in wire. Tyr383, which is within hydrogen bond distance to the benzimidazole nitrogen of the ligand, is displayed in stick for reference

For family A compounds, the use of S_Comp_ turned out to be not strictly necessary. If the top pose for each compound was selected based on S_Comp_, the median and maximum RMSD were 0.8 and 2.0 Å, respectively. If ICM docking score S_Dock_ was used for selection, the median and maximum RMSD were 1.0 and 2.0 Å, respectively. Both scores would correctly select ligand poses that are within RMSD of 2.0 Å. Figure 3 shows the predicted docking pose of a representative member FXR_26 versus its co-crystallized X-ray structure, with an RMSD of 0.3 Å. The success of pose prediction by ICM docking score alone for this family is partly due to the fact that similar benzimidazole compounds have been co-crystallized in seven PDBs (PDB ID: 3OKI, 3OKH, 3OMK, 3OOF, 3OLF, 3OMM, and 3OOK) in the Pocketome entry used for docking. Figure 3 shows that not only the predicted ligand pose, but the protein conformation selected by the docking procedure for FXR_26 is very similar to the released structure, having a ligand pocket backbone C_α_ RMSD of 0.2 Å.

**Fig. 4:**
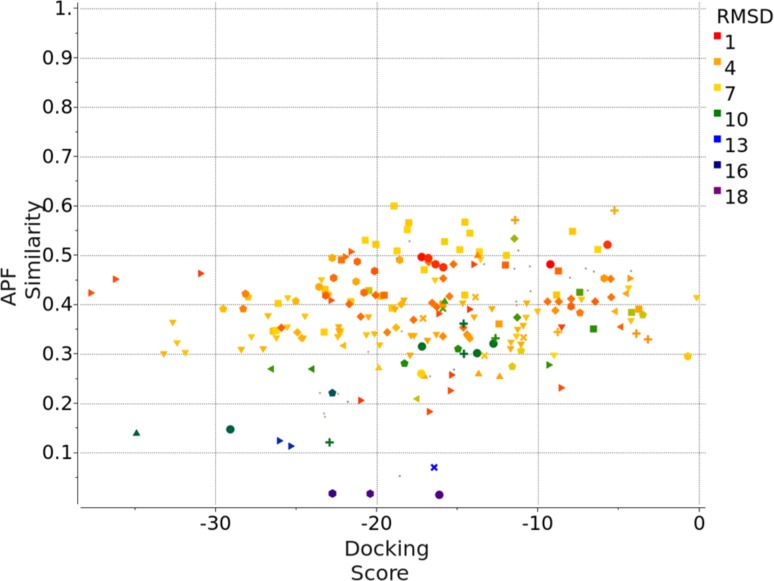
APF Similarity score versus ICM Docking Score for three Family B compounds containing the spiro core, docked to 40 protein conformations in Pocketome entry NR1H4_HUMAN_257_485, each in two independent runs: each compound generated 80 poses. Only the poses that have ICM docking score <0 are shown here. The pairwise APF distance between each pose were calculated, the poses were then clustered at an APF distance cutoff of 0.4. Poses belonging to the same cluster were represented by the same shape. After the release of the X-ray structure, each predicted pose’s RMSD to the correct pose was calculated and shown in *color gradient* according to the RMSD value

**Fig. 5:**
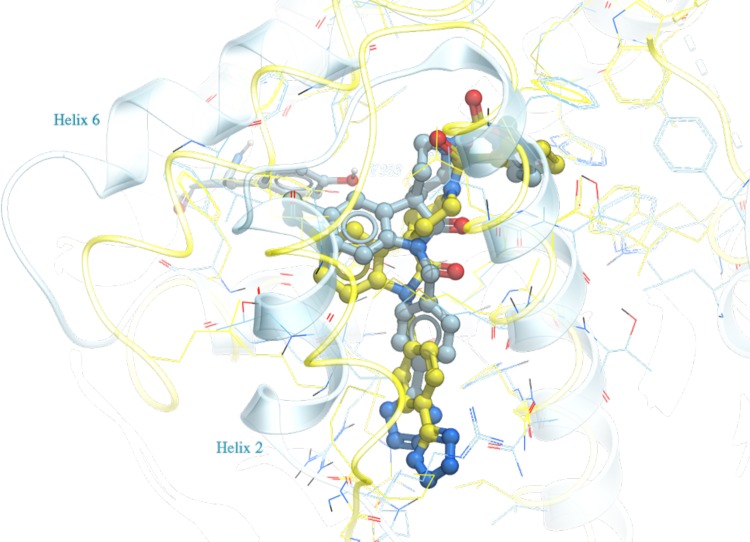
Predicted docking pose for FXR_11 (*blue*) from family B versus the co-crystallized structure released (*yellow*). All residues within 5 Å of the ligand are displayed in wire. Tyr383 is displayed in stick and labeled for reference. Helices 2 and 6, on the *bottom and top left*, are labeled, and shown major displacement between the PDB used for docking (3FLI) and the solved X-ray structure

Compound family B has a spiro[indoline-3,4′-piperidin]-2-one moiety at its core, containing three members out of the first 36 compounds, and 22 members out of the full set of 102 compounds. Figure 4 is the plot of APF similarity score versus ICM docking score for the three family B compounds. The cluster of poses that has the best composite score S_Comp_, and eventually shown to have the best RMSD, is at the top left portion of the plot. For family B, the maximum APF similarity score is around 0.4–0.6, because this class of spiro compounds has not been previously co-crystallized in the Pocketome PDB entries. Unlike family A, in which the top pose cluster shows a clear separation from the rest of the poses in terms of ICM docking score, in family B there are two pose families that have somewhat comparable docking score. Figure 5 shows the top pose of a representative member, FXR_11, selected by the composite score S_Comp_. The predicted pose has a RMSD of 2.1 Å versus the X-ray structure. The PDB selected for docking of FXR_11 (PDB code: 3FLI) has a ligand pocket backbone C_α_ RMSD of 2.1 Å versus the released structure. As seen in Fig. 5, there are major displacements in helices 2 and 6 on one side of the ligand pocket, resulting in a borderline correct, but laterally shifted docking pose of the ligand.

**Fig. 6:**
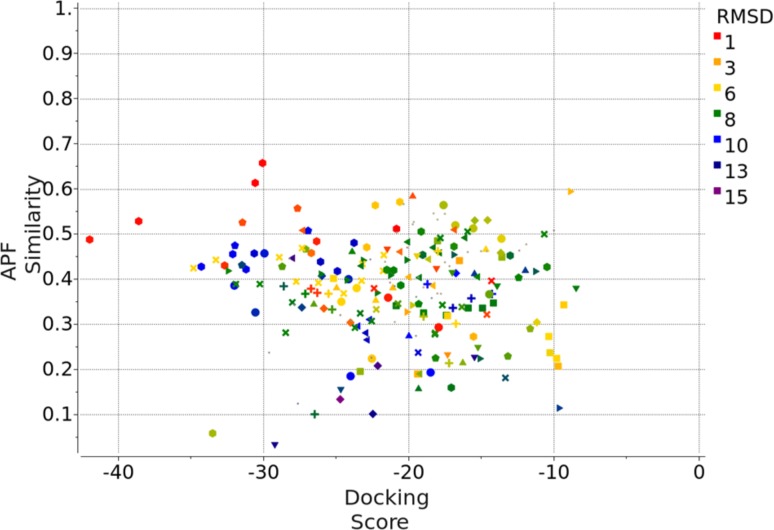
APF Similarity score versus ICM Docking Score for three Family C compounds containing either 4,5,6,7-tetrahydro-1*H*-pyrrolo[2,3-c]pyridine or 4,5,6,7-tetrahydro-3*H*-pyrazolo[4,3-c]pyridine core, docked to 40 protein conformations in Pocketome entry NR1H4_HUMAN_257_485, each in two independent runs: each compound generated 80 poses. The pairwise APF distance between each pose were calculated, the poses were then clustered at an APF distance cutoff of 0.4. Poses belonging to the same cluster were represented by the same shape. After the release of the X-ray structure, each predicted pose’s RMSD to the correct pose was calculated and shown in *color gradient* according to the RMSD value

**Fig. 7:**
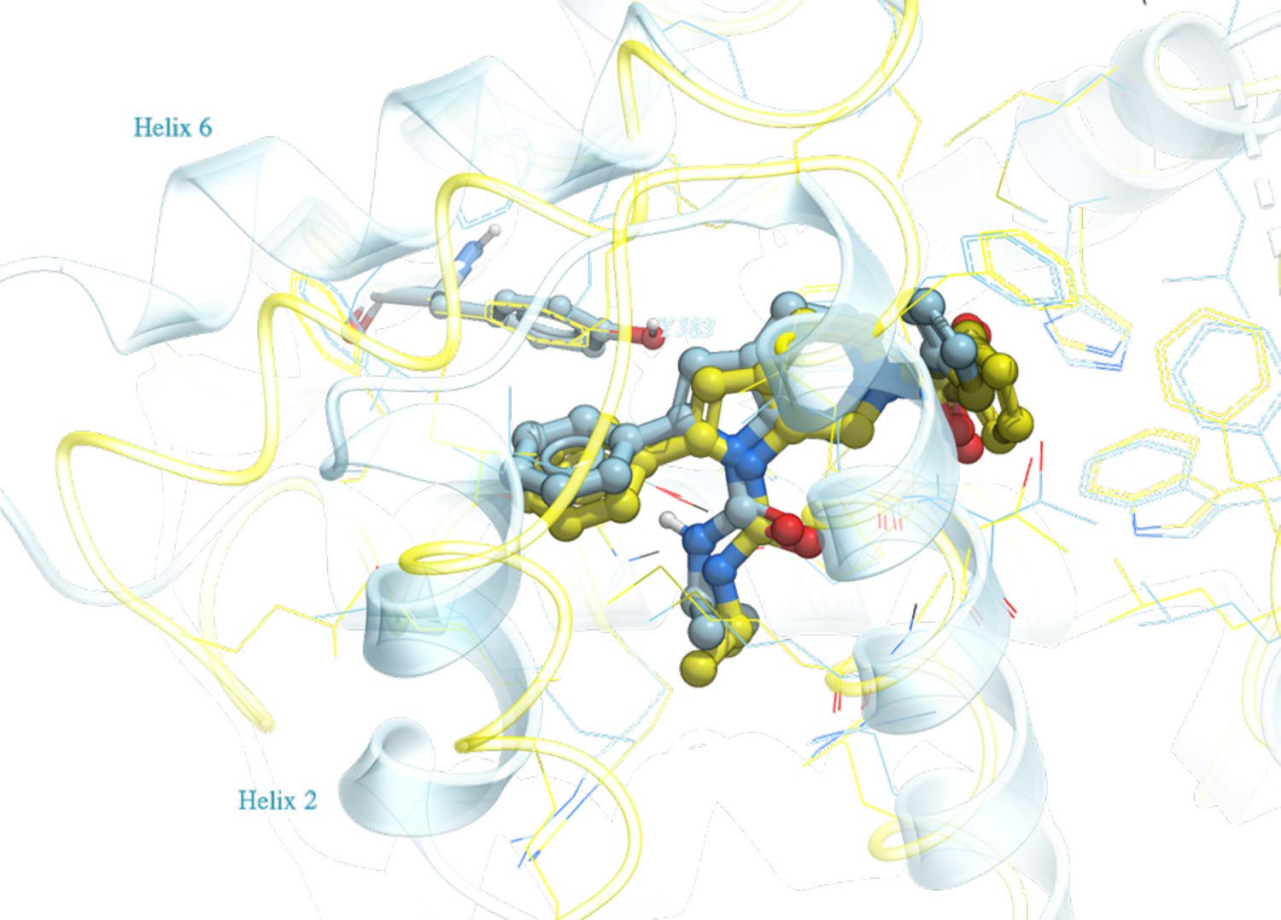
Predicted docking pose for FXR_16 (*blue*) from family B versus the co-crystallized structure released (*yellow*). All residues within 5 Å of the ligand are displayed in *wire*. Tyr383 is displayed in stick and labeled for reference. Helices 2 and 6, on the *bottom and top left*, are labeled, and shown major displacement between the PDB used for docking (3FLI) and the solved X-ray structure

Compound family C contains 3 members out of the first 36 compounds, and 23 members out of the full set of 102 compounds. It can be broken down further into two sub-families, one containing the 4,5,6,7-tetrahydro-1*H*-pyrrolo[2,3-c]pyridine core, the other containing the 4,5,6,7-tetrahydro-3*H*-pyrazolo[4,3-c]pyridine core. Figure 6 is the plot of APF similarity score versus ICM docking score for the three family C compounds. The cluster of poses that has the best composite score S_Comp_ and the best RMSD, is again at the top left portion of the plot. Note that family C compound has not been previously co-crystallized in the Pocketome PDB entries, therefore the maximum APF similarity to co-crystallized ligands is around 0.5–0.6. Also note that for one of the members, FXR_15, the top pose according to S_Comp_ is an incorrect one. However, by clustering all poses using the APF method, and selecting the pose with the best S_Comp_ within each pose cluster, we were able to identify the correct pose within the top five ranked poses. Figure 7 shows the top pose of a representative member, FXR_16, selected by the composite score S_Comp_. The predicted pose has a RMSD of 1.3 Å versus the X-ray structure. For FXR_16, the PDB selected for docking (PDB code: 3FLI) has a ligand pocket backbone C_α_ RMSD of 1.8 Å versus the released structure. Again, there are major displacements in helices 2 and 6 on one side of the ligand pocket, but they do not appear to adversely affect the accuracy of docking.

**Fig. 8:**
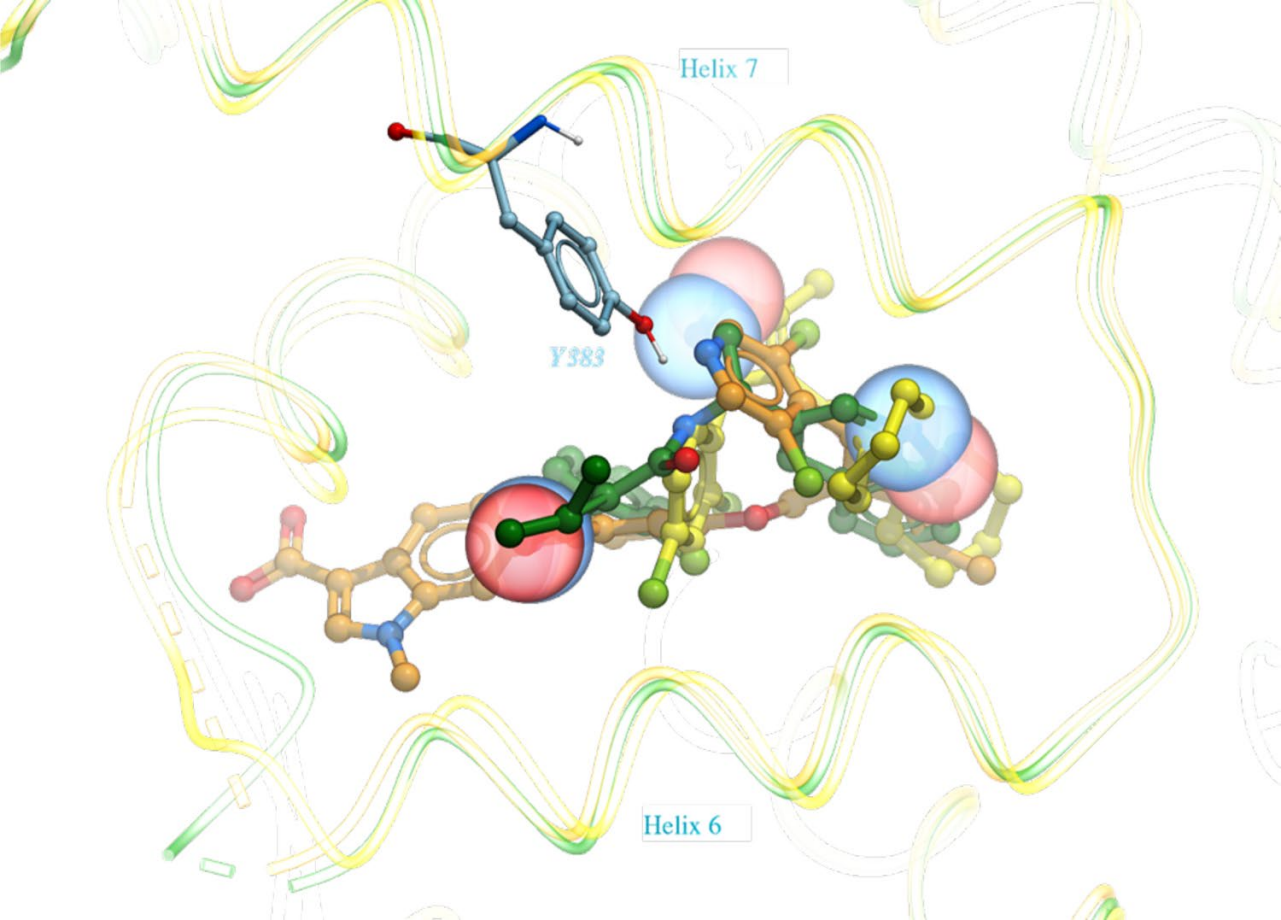
The released X-ray structures for FXR_4 (*yellow*), FXR_23 (*green*), and FXR_33 (*orange*) from family D. The oxygen and nitrogen atoms of the isoxazole core common in all three compounds are displayed in CPK representation. Tyr383 is displayed in stick and labeled for reference. Helices 6 and 7 are labeled

The docking results for all 36 compounds are shown in Supplementary Table 1. For the top pose that we have selected for each compound based solely on S_Comp_, 14 out of 36 were docked within 1 Å RMSD, 24 out of 36 were within 2 Å. Three Compounds, all from the family B spiro class, were docked between 2.0 and 2.6 Å. For FXR_34, a steroidal compound with long flexible substituent, the best pose was found at rank 4, with RMSD of 1.6 Å. Out of the 36 compounds, only six (FXR_1, FXR_2, FXR_3, FXR_4, FXR_18, FXR_23) were docked incorrectly among all of the top five ranked poses. Out of these six mis-docked compounds, two of them belong to family D containing an isoxazole. While we docked FXR_33 from family D correctly at 0.2 Å RMSD, FXR_4 and FXR_23 were mis-docked. Figure 8 shows the released X-ray structures for these compounds; the isoxazole groups in all three compounds occupy different space within the ligand pocket. This group of ligands illustrates the limitations of the assumption underlying the ligand-biased approach, i.e. that chemically similar moieties should form similar receptor interactions.

**Fig. 9:**
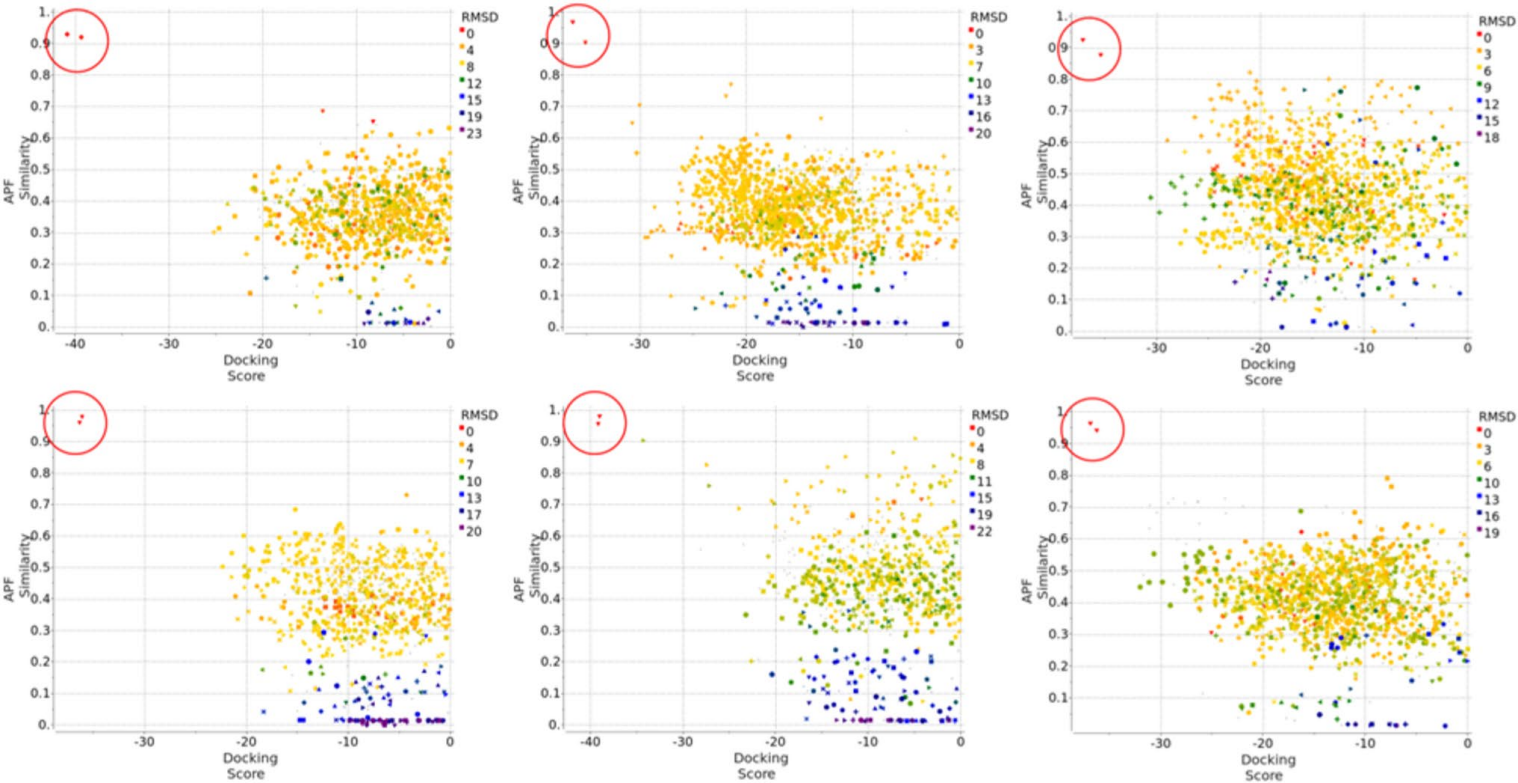
APF Similarity score versus ICM Docking Score. *Top left to right* FXR_1, FXR2, and FXR_3; *bottom left to right* FXR_4, FXR_18, and FXR_23. The plot combines docking results of: 1. 40 protein conformations in Pocketome entry NR1H4_HUMAN_257_485, each in two independent runs, both with ligand APF bias, top 10 poses were retained. 2. 36 newly released X-ray structure, each in two independent runs, one with co-crystallized ligand APF template, one without, top 10 poses were retained. Only the poses that have ICM docking score <0 are shown here. The pairwise APF distance between each pose were calculated, the poses were then clustered at an APF distance cutoff of 0.4. Poses belonging to the same cluster were represented by the same shape. Each predicted pose’s RMSD to the correct pose was calculated and shown in *color gradient* according to the RMSD value. The two poses that came from the docking to the native X-ray structure, one with ligand APF template, one without, were *circled in red*

To investigate the reason we failed to dock the six compounds correctly using the available Pocketome structures, we re-evaluated the docking results to the 40 Pocketome structures by taking the top 10 docking poses for each of the two independent runs, both with ligand APF template, calculated their ICM docking score, APF similarity to the corresponding co-crystallized ligand, each compound generated 800 poses from the 40 Pocketome entries. In addition, we also docked each of these six compounds to each of the 36 newly released X-ray structures, one of which is the cognate structure for that compound. Two independent runs were performed, one with a co-crystallized ligand APF template, one without. The reason was to ascertain if a co-crystallized ligand used as APF template during docking is necessary, or if a correct protein conformation is sufficient for cognate receptor structure docking. The top 10 poses for each independent run were reevaluated by ICM docking score and APF Similarity score calculations. Combining the docking results to the 40 Pocketome entries and to the 36 newly released structures produced 1520 poses for each compound. Figure 9 is the plot of APF similarity score versus ICM docking score for the six mis-docked compounds. Each compound showed two near-native docking solutions that have the best ICM docking score, APF similarity score, and ligand RMSD. These two docking solutions originated from the docking to the corresponding cognate structure, with or without co-crystallized ligand APF bias during docking. We can conclude that: (1) When cognate receptor structure is used, ICM docking can find near native pose for each of the six ligands, with or without use of an APF template. (2) However, none of the available non-cognate X-ray structures presented a receptor conformation that resembles sufficiently the conformations induced by the six ligands to allow near-native pose generation even beyond the top 5 poses, and no crystallographic ligand was useful as a biasing template.

**Fig. 10:**
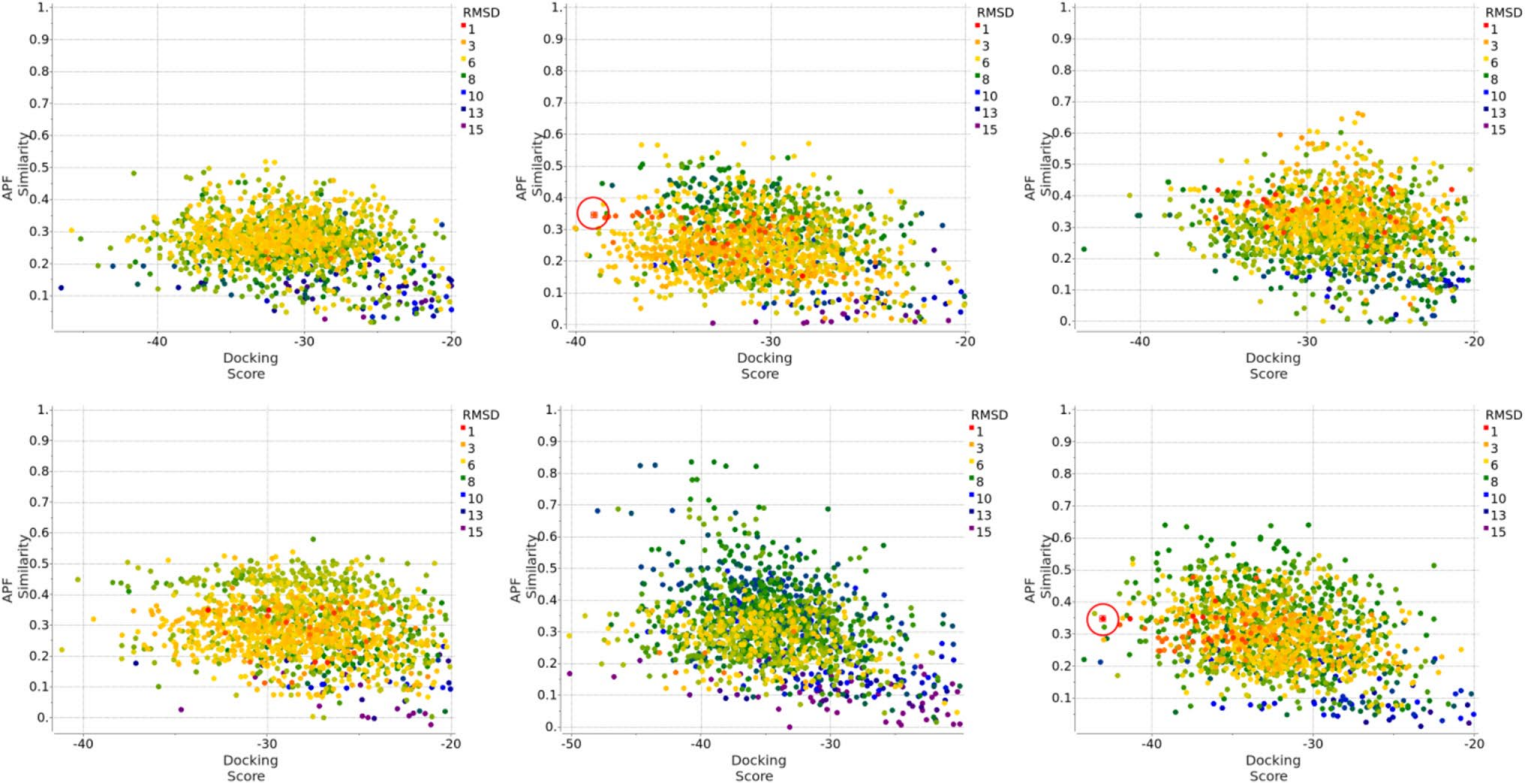
APF Similarity score versus ICM Docking Score. *Top left to right* FXR_1, FXR2, and FXR_3; *bottom left to right* FXR_4, FXR_18, and FXR_23. The plot shows the result of SCARE simulation followed by fully flexible side chain refinement. For FXR_2 and FXR_23, near native solutions with 2.3 and 1.1 Å RMSD, respectively, were found in the top five poses according to ICM docking score

To investigate if generating additional protein conformations would have helped finding the correct docking pose, we carried out SCARE simulations [23] on each of the 6 mis-docked compounds starting with each of the 40 Pocketome conformations (i.e. using the same X-ray data as available during the challenge but allowing new receptor conformation generation via SCARE protocol). In each run, the top 40 conformations were retained, followed by fully flexible side chain refinement. For each of the six compounds we generated a total of 1600 poses. The plot of APF similarity to the co-crystallized ligand versus ICM docking score is shown in Fig. 10. For the six compounds FXR_1, FXR_2, FXR_3, FXR_4, FXR_18 and FXR_23, the lowest RMSD achieved in each stack of 1600 poses are 2.7, 1.2, 1.4, 0.8, 2.6, and 1.1 Å, respectively. Thus SCARE protocol is capable of, at least, generating good quality near-native poses for four out of six difficult cases. It should be noted that the remaining ligands FXR_1 and FXR_18 are two of the most difficult compounds for this docking challenge, as none of the GC2 submissions achieved better than 3.0 Å RMSD. Two of the six compounds would make it into the five top-scored solutions: for FXR_2, a near native pose of 2.3 Å RMSD is found as the fifth top scoring pose and for FXR_23, a native pose of 1.1 Å RMSD is found as the fourth top scoring pose. We also clustered each set of 1600 poses at an APF cutoff distance of 0.4, the total number of clustered poses for the six compounds are 613, 453, 561, 447, 625, and 514, respectively; the lowest RMSD after clustering are 2.7, 1.8, 1.9, 2.0, 2.8, and 1.1 Å, respectively. Thus, extensive binding site flexibility sampling can indeed generate near-native poses for these difficult cases but consistent identification/ranking of such poses among a variety of non-native solutions presents a challenge.

## Conclusions

In this docking assessment we successfully docked 14 out of 36 compounds to be within atomic resolution accuracy, 30 out of 36 compounds were docked correctly depending on measuring metrics. Using multiple experimentally resolved receptor conformations was essential to correctly reproduce bound poses across multiple ligands chemotypes. Another important factor in the successful prediction was the use of APF methodology to improve docking and pose selection in three ways: (1) Use of co-crystallized ligand APF bias during docking. (2) Use of composite score that combines APF 3D chemical similarity score to cognate ligand with ICM docking score in post-docking pose selection. (3) Use of clustering based on APF 3D chemical similarity to group compounds from the same chemical class to check for the consistency in docking poses. Further improvements can be made in the future by generating alternative protein conformations not available in the experimental structures, but challenges remain in pose scoring and selection.

## Electronic supplementary material

Below is the link to the electronic supplementary material.

Supplementary material 1 (PDF 217 KB)

## Abbreviations

PDB: Protein Data Bank
APF: Atomic property field
