# Supplementary material for: Ligand-biased ensemble receptor docking (LigBEnD): a hybrid ligand/receptor structure-based approach
Source: J Comput Aided Mol Des. 2017 Sep 8;32(1):187–98. doi: 10.1007/s10822-017-0058-x (PMC5767200; doi:10.1007/s10822-017-0058-x)
Supplement: Supplementary file 1 — Supplementary material 1 (PDF 217 KB) [file 10822_2017_58_MOESM1_ESM.pdf]

Suppl. Table 1

Compound Structure[a]	Compound ID	PDB used for Docking	Ligand RMSD for top ranked (Å)[b]	C_α RMSD for top ranked (Å)[c]	Minimum Ligand RMSD for top 5 ranked (Å)
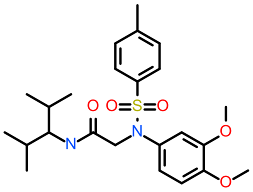	FXR_1	4OIV	5.6	5.6	5.2
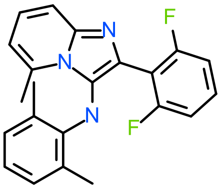	FXR_2	4OIV	4.1	3.1	4.0
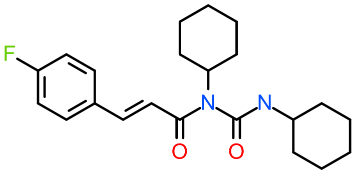	FXR_3	3OKI	3.5	3.8	3.4
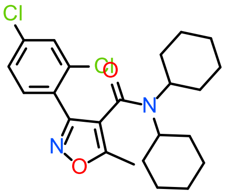	FXR_4	3OKI	7.0	2.7	3.9
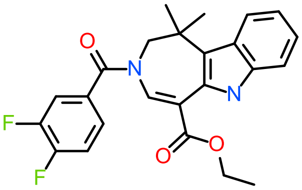	FXR_5	3FLI	0.5	0.9	0.5

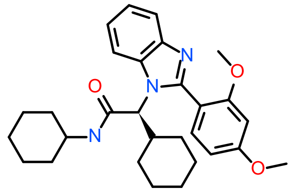	FXR_6	3OKI	1.6	0.2	1.6
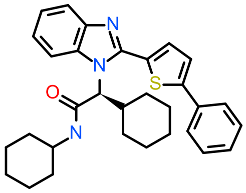	FXR_7	3OMK	0.6	0.4	0.6
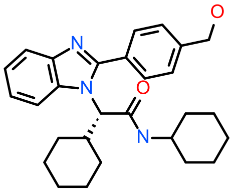	FXR_8	3OKI	0.3	0.2	0.3
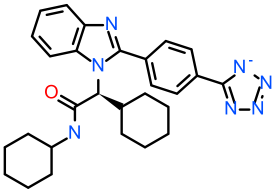	FXR_9	3OKI	0.2	0.2	0.2
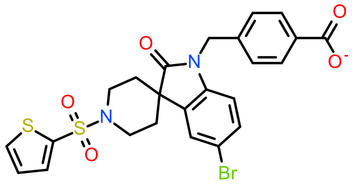	FXR_10	3FLI	2.5	2.2	2.5
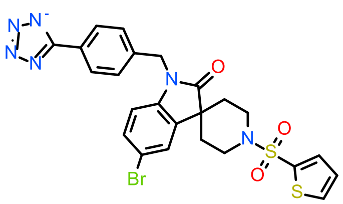	FXR_11	3FLI	2.1	2.1	2.1

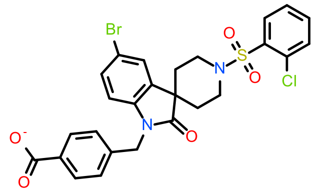	FXR_12	3FLI	2.6	2.3	2.6
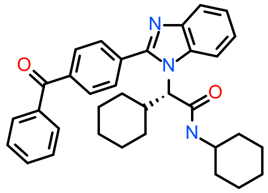	FXR_13	3OMK	0.4	0.5	0.4
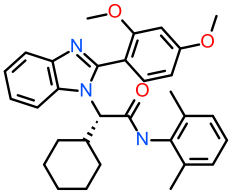	FXR_14	3OMK	1.6	0.4	1.1
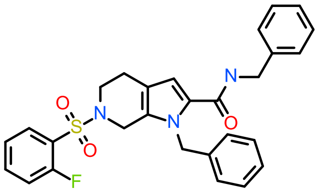	FXR_15	3FXV	11.0	0.7	1.8
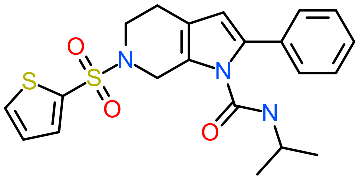	FXR_16	3FLI	1.3	1.8	1.3
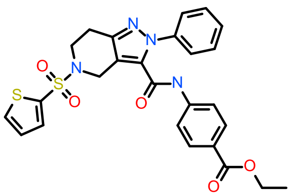	FXR_17	3FLI	1.5	1.8	1.5

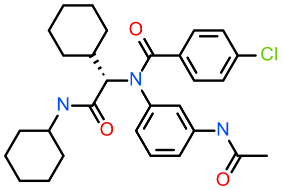	FXR_18	3OKH	9.0	1.7	7.4
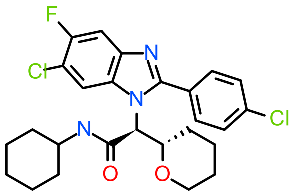	FXR_19	3OKI	1.1	2.3	1.1
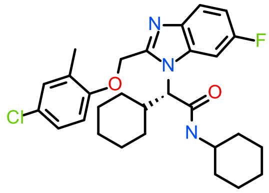	FXR_20	3OKI	0.6	0.3	0.6
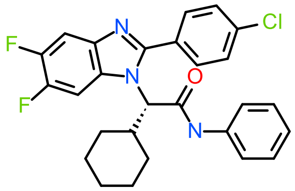	FXR_21	3OOF	0.8	0.2	0.8
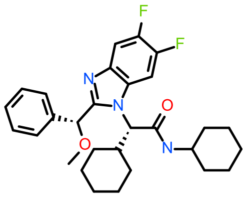	FXR_22	3OKI	1.9	0.4	1.9
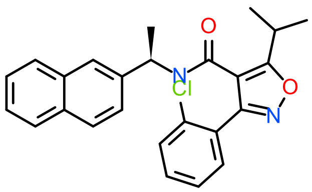	FXR_23	3RVF	8.0	2.1	5.7

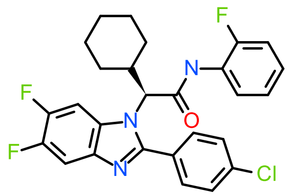	FXR_24	3OMK	1.2	0.1	1.2
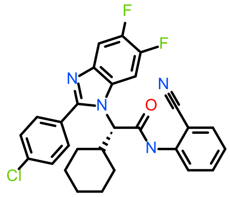	FXR_25	3OMK	0.3	0.2	0.3
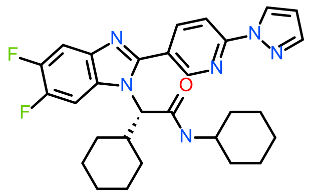	FXR_26	3OKI	0.3	0.2	0.3
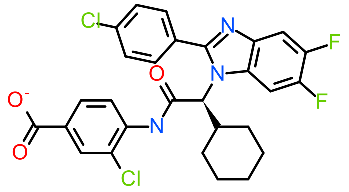	FXR_27	3OOK	0.3	0.4	0.3
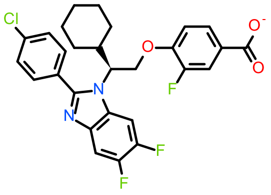	FXR_28	3OOK	1.2	0.3	1.2
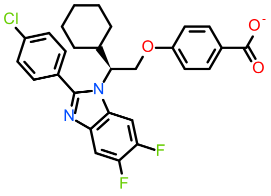	FXR_29	3OOK	0.8	0.3	0.8

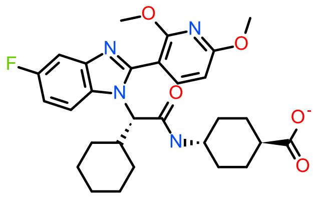	FXR_30	3OLF	1.6	0.3	1.5
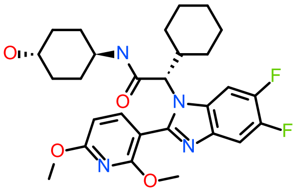	FXR_31	3OKI	1.5	0.3	1.5
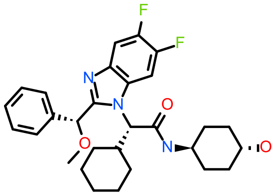	FXR_32	3OKI	2.1	0.5	2.1
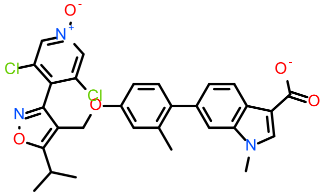	FXR_33	3FXV	0.2	0.2	0.2
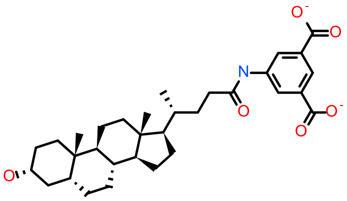	FXR_34	1OSV	3.5	2.3	1.6
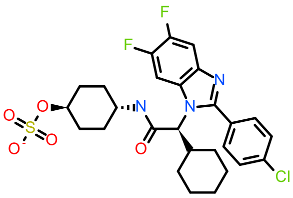	FXR_35	3OKI	0.5	0.3	0.5

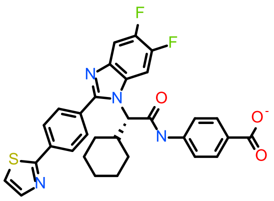	FXR_36	3OMM	0.7	0.5	0.7

Table 1. Docking results of the 36 compounds.

[a] Structure was converted converted in ICM from SMILES supplied by assessment organizer, charge state was set by pKa predictor in ICM at pH 7.

[b] The protein conformation used in docking and the correct structure was first superimposed by C_α of the residues within 7 Å radius of the ligand. Only the non-hydrogen atoms of the ligands were used in RMSD calculation.

[c] Only the C_α atoms of the residues within 5 Å radius of the ligands were used for RMSD calculations.

AUTHOR INFORMATION

Corresponding Author

*E-mail: max@molsoft.com

Author Contributions

The manuscript was written through contributions of all authors. All authors have given approval to the final version of the manuscript.

Notes

The authors declare no competing financial interest.

ACKNOWLEDGMENT

The authors thank D3R organizers for coordinating the challenge. We also thank Eugene Raush for technical assistance, and Andrew Orry for proofreading of this manuscript.

ABBREVIATIONS

PDB, Protein Data Bank; APF, Atomic Property Field.

REFERENCES

1. Bottegoni, G.; Rocchia, W.; Rueda, M.; Abagyan, R.; Cavalli, A., Systematic exploitation of multiple receptor conformations for virtual ligand screening. *PLoS One* **2011,** *6* (5), e18845.

2. Repasky, M. P.; Murphy, R. B.; Banks, J. L.; Greenwood, J. R.; Tubert-Brohman, I.; Bhat, S.; Friesner, R. A., Docking performance of the glide program as evaluated on the Astex and DUD datasets: a complete set of glide SP results and selected results for a new scoring function integrating WaterMap and glide. *J Comput Aided Mol Des* **2012,** *26* (6), 787-99.

3. Spitzer, R.; Jain, A. N., Surflex-Dock: Docking benchmarks and real-world application. *J Comput Aided Mol Des* **2012,** *26* (6), 687-99.

4. Morris, G. M.; Huey, R.; Lindstrom, W.; Sanner, M. F.; Belew, R. K.; Goodsell, D. S.; Olson, A. J., AutoDock4 and AutoDockTools4: Automated docking with selective receptor flexibility. *J Comput Chem* **2009,** *30* (16), 2785-91.

5. Ruiz-Carmona, S.; Alvarez-Garcia, D.; Foloppe, N.; Garmendia-Doval, A. B.; Juhos, S.; Schmidtke, P.; Barril, X.; Hubbard, R. E.; Morley, S. D., rDock: a fast, versatile and open source program for docking ligands to proteins and nucleic acids. *PLoS Comput Biol* **2014,** *10* (4), e1003571.

6. Allen, W. J.; Balius, T. E.; Mukherjee, S.; Brozell, S. R.; Moustakas, D. T.; Lang, P. T.; Case, D. A.; Kuntz, I. D.; Rizzo, R. C., DOCK 6: Impact of new features and current docking performance. *J Comput Chem* **2015,** *36* (15), 1132-56.

7. Venkatachalam, C. M.; Jiang, X.; Oldfield, T.; Waldman, M., LigandFit: a novel method for the shape-directed rapid docking of ligands to protein active sites. *J Mol Graph Model* **2003,** *21* (4), 289-307.

8. Friesner, R. A.; Banks, J. L.; Murphy, R. B.; Halgren, T. A.; Klicic, J. J.; Mainz, D. T.; Repasky, M. P.; Knoll, E. H.; Shelley, M.; Perry, J. K.; Shaw, D. E.; Francis, P.; Shenkin, P. S., Glide: a new approach for rapid, accurate docking and scoring. 1. Method and assessment of docking accuracy. *J Med Chem* **2004,** *47* (7), 1739-49.

9. Jones, G.; Willett, P.; Glen, R. C.; Leach, A. R.; Taylor, R., Development and validation of a genetic algorithm for flexible docking. *J Mol Biol* **1997,** *267* (3), 727-48.

10. Corbeil, C. R.; Williams, C. I.; Labute, P., Variability in docking success rates due to dataset preparation. *J Comput Aided Mol Des* **2012,** *26* (6), 775-86.

11. Jain, A. N., Surflex: fully automatic flexible molecular docking using a molecular similarity-based search engine. *J Med Chem* **2003,** *46* (4), 499-511.

12. Plewczynski, D.; Łaźniewski, M.; Augustyniak, R.; Ginalski, K., Can we trust docking results? Evaluation of seven commonly used programs on PDBbind database. *J Comput Chem* **2011,** *32* (4), 742-55.

13. Wang, Z.; Sun, H.; Yao, X.; Li, D.; Xu, L.; Li, Y.; Tian, S.; Hou, T., Comprehensive evaluation of ten docking programs on a diverse set of protein-ligand complexes: the prediction accuracy of sampling power and scoring power. *Phys Chem Chem Phys* **2016,** *18* (18), 12964-75.

14. Warren, G. L.; Andrews, C. W.; Capelli, A. M.; Clarke, B.; LaLonde, J.; Lambert, M. H.; Lindvall, M.; Nevins, N.; Semus, S. F.; Senger, S.; Tedesco, G.; Wall, I. D.; Woolven, J. M.; Peishoff, C. E.; Head, M. S., A critical assessment of docking programs and scoring functions. *J Med Chem* **2006,** *49* (20), 5912-31.

15. Cross, J. B.; Thompson, D. C.; Rai, B. K.; Baber, J. C.; Fan, K. Y.; Hu, Y.; Humblet, C., Comparison of several molecular docking programs: pose prediction and virtual screening accuracy. *J Chem Inf Model* **2009,** *49* (6), 1455-74.

16. Carlson, H. A.; Smith, R. D.; Damm-Ganamet, K. L.; Stuckey, J. A.; Ahmed, A.; Convery, M. A.; Somers, D. O.; Kranz, M.; Elkins, P. A.; Cui, G.; Peishoff, C. E.; Lambert, M. H.; Dunbar, J. B., CSAR 2014: A Benchmark Exercise Using Unpublished Data from Pharma. *J Chem Inf Model* **2016,** *56* (6), 1063-77.

17. Neves, M. A.; Totrov, M.; Abagyan, R., Docking and scoring with ICM: the benchmarking results and strategies for improvement. *J Comput Aided Mol Des* **2012,** *26* (6), 675-86.

18. Husby, J.; Bottegoni, G.; Kufareva, I.; Abagyan, R.; Cavalli, A., Structure-based predictions of activity cliffs. *J Chem Inf Model* **2015,** *55* (5), 1062-76.

19. Durrant, J. D.; McCammon, J. A., Molecular dynamics simulations and drug discovery. *BMC Biol* **2011,** *9*, 71.

20. Fukunishi, Y.; Mashimo, T.; Misoo, K.; Wakabayashi, Y.; Miyaki, T.; Ohta, S.; Nakamura, M.; Ikeda, K., Miscellaneous Topics in Computer-Aided Drug Design: Synthetic Accessibility and GPU Computing, and Other Topics. *Curr Pharm Des* **2016,** *22* (23), 3555-68.

21. Totrov, M.; Abagyan, R., Flexible ligand docking to multiple receptor conformations: a practical alternative. *Curr Opin Struct Biol* **2008,** *18* (2), 178-84.

22. Rueda, M.; Bottegoni, G.; Abagyan, R., Recipes for the selection of experimental protein conformations for virtual screening. *J Chem Inf Model* **2010,** *50* (1), 186-93.

23. Bottegoni, G.; Kufareva, I.; Totrov, M.; Abagyan, R., A new method for ligand docking to flexible receptors by dual alanine scanning and refinement (SCARE). *J Comput Aided Mol Des* **2008,** *22* (5), 311-25.

24. Rueda, M.; Totrov, M.; Abagyan, R., ALiBERO: evolving a team of complementary pocket conformations rather than a single leader. *J Chem Inf Model* **2012,** *52* (10), 2705-14.

25. Warszycki, D.; Rueda, M.; Mordalski, S.; Kristiansen, K.; Satała, G.; Rataj, K.; Chilmonczyk, Z.; Sylte, I.; Abagyan, R.; Bojarski, A. J., From Homology Models to a Set of Predictive Binding Pockets-a 5-HT1A Receptor Case Study. *J Chem Inf Model* **2017,** *57* (2), 311-321.

26. Bottegoni, G.; Kufareva, I.; Totrov, M.; Abagyan, R., Four-dimensional docking: a fast and accurate account of discrete receptor flexibility in ligand docking. *J Med Chem* **2009,** *52* (2), 397-406.

27. Burley, S. K.; Berman, H. M.; Kleywegt, G. J.; Markley, J. L.; Nakamura, H.; Velankar, S., Protein Data Bank (PDB): The Single Global Macromolecular Structure Archive. *Methods Mol Biol* **2017,** *1607*, 627-641.

28. Totrov, M., Atomic property fields: generalized 3D pharmacophoric potential for automated ligand superposition, pharmacophore elucidation and 3D QSAR. *Chem Biol Drug Des* **2008,** *71* (1), 15-27.

29. Chen, Y. C.; Totrov, M.; Abagyan, R., Docking to multiple pockets or ligand fields for screening, activity prediction and scaffold hopping. *Future Med Chem* **2014,** *6* (16), 1741-55.

30. Grigoryan, A. V.; Kufareva, I.; Totrov, M.; Abagyan, R. A., Spatial chemical distance based on atomic property fields. *J Comput Aided Mol Des* **2010,** *24* (3), 173-82.

31. Kufareva, I.; Ilatovskiy, A. V.; Abagyan, R., Pocketome: an encyclopedia of small-molecule binding sites in 4D. *Nucleic Acids Res* **2012,** *40* (Database issue), D535-40.

32. Abagyan, R.; Totrov, M., Biased probability Monte Carlo conformational searches and electrostatic calculations for peptides and proteins. *J Mol Biol* **1994,** *235* (3), 983-1002.

33. Orry, A. J.; Abagyan, R., Preparation and refinement of model protein-ligand complexes. *Methods Mol Biol* **2012,** *857*, 351-73.

34. Arnautova, Y. A.; Abagyan, R. A.; Totrov, M., Development of a new physics-based internal coordinate mechanics force field and its application to protein loop modeling. *Proteins* **2011,** *79* (2), 477-98.

35. Katritch, V.; Totrov, M.; Abagyan, R., ICFF: a new method to incorporate implicit flexibility into an internal coordinate force field. *J Comput Chem* **2003,** *24* (2), 254-65.

36. Totrov, M.; Abagyan, R., Flexible protein-ligand docking by global energy optimization in internal coordinates. *Proteins* **1997,** *Suppl 1*, 215-20.
